# Optimising Transformation Efficiency in *Borrelia:* Unravelling the Role of the Restriction-Modification System of *Borrelia afzelii* and *Borrelia garinii*

**DOI:** 10.3390/ijms252111343

**Published:** 2024-10-22

**Authors:** Margarida Ruivo, Noémi Zsuzsa Kovács, Anna-Margarita Schötta, Theresa Stelzer, Laura Hermann, Verena Mündler, Andreas Bergthaler, Michael Reiter, Michiel Wijnveld

**Affiliations:** 1 Institute for Hygiene and Applied Immunology, Center for Pathophysiology, Infectiology and Immunology, Medical University of Vienna, Kinderspitalgasse 15, 1090 Vienna, Austria; margarida.ruivo@meduniwien.ac.at (M.R.); nzsk.microbio22@gmail.com (N.Z.K.); anna.schoetta@gmx.at (A.-M.S.); thestelzer.molbio@outlook.com (T.S.); laura.hermann04@gmail.com (L.H.); enamue@gmail.com (V.M.); andreas.bergthaler@meduniwien.ac.at (A.B.); michael.a.reiter@meduniwien.ac.at (M.R.); 2 CeMM Research Center for Molecular Medicine, Austrian Academy of Sciences, Lazarettgasse 14 AKH BT 25.3, 1090 Vienna, Austria

**Keywords:** *Borrelia burgdorferi* sensu lato, endonuclease, genetic transformation efficiency, methyltransferase, restriction-modification system

## Abstract

*Borrelia* spp. are transmitted to humans by the bite of an infected tick. In Europe, *Borrelia afzelii* and *Borrelia garinii* are the main causative agents of Lyme borreliosis, one of the most prevalent tick-borne diseases in the northern hemisphere. In bacteria such as *Borrelia* spp., a restriction-modification system (RMS) protects against the harmful introduction of foreign DNA. The RMS comprises two activities: methyltransferase and endonuclease. This study is aimed to characterize the RMS of *B. afzelii* and *B. garinii*. First, we identified potential RMS genes. The predicted genes were cloned into a methylase-deficient *Escherichia coli* strain and digested with methylation-sensitive restriction enzymes to verify methyltransferase activity. Additionally, the RMS proteins were purified to evaluate endonuclease activity. Subsequently, methylated and unmethylated plasmids were used to investigate the effect of methylation on endonuclease activity and transformation efficiency. We identified four possible RMS genes in *B. afzelii* and four RMS genes in *B. garinii*. We analyzed the presence of these genes in patient isolates and observed a high degree of heterogeneity. The restriction pattern of DNA methylated by each of the four recombinantly expressed genes provided strong evidence that all encode adenine-specific methyltransferases. After 24 h of incubation with purified RMS proteins, we observed complete digestion of unmethylated plasmid DNA, demonstrating endonuclease activity. Finally, we proved that methylation protects against endonuclease activity and increases transformation efficiency.

## 1. Introduction

Lyme borreliosis is one of Europe’s most prevalent tick-borne diseases, with more than 200,000 cases annually in Western Europe [1]. The principal causative agents of this disease in Europe are members of the *Borrelia burgdorferi* sensu lato (*Bbsl*) species complex (e.g., *Borrelia afzelii, Borrelia garinii* and *Borrelia burgdorferi* sensu stricto (*Bbss*)) that are transmitted by *Ixodes* spp. ticks [2].

*Borrelia* is a genus of bacteria of the phylum Spirochaetes whose natural infectious cycle alternates between tick vectors and vertebrate hosts such as rodents or birds and with humans as dead-end hosts [2,3]. The genome of these spirochetes is unique and complex, consisting of a linear chromosome of approximately 900 kb and over 20 linear (lp) and circular (cp) plasmids [4]. Currently, molecular investigation tools are only available for high-passage laboratory strains [5,6]. The plasmid profile of *Borrelia* spp. is relatively unstable during in vitro propagation [7,8]. In a study by Grimm et al. [9] published in 2004, loss of linear plasmids lp25, lp28-1, and lp36 in *B. burgdorferi* sensu stricto (*Bbss*) strain B31 occurred during in vitro cultivation, ultimately resulting in a loss of infectivity in mice. Therefore, *Bbss* B31 is considered a high passage strain with fewer plasmids, while low passage strains have more plasmids due to limited cultivation [10]. Furthermore, it was observed that in *Bbss* the loss of lp25 and lp56 results in significantly increased transformation efficiency using shuttle vectors, suggesting that these plasmids contained genes providing protection against the introduction of foreign DNA. Computational analysis revealed genes *bbe02* in lp25 and *bbq67* in lp56, which were assumed to be part of a restriction-modification system (RMS) [11]. Several studies have found that *Borrelia* can lose the ability to infect and cause disease after repeated in vitro passage [12]. This suggests that low-passage strains contain several proteins that are absent or expressed in smaller quantities in high-passage strains, including the RMS [5,12].

The RMS is a primary defense mechanism against bacteriophage infection and other invading DNA elements. A typical RMS comprises two distinct enzymatic activities: a restriction endonuclease activity that cleaves the phosphodiester bonds of the DNA backbone and a methyltransferase activity that catalyzes the addition of a methyl group to DNA adenine or cytosine. Both enzymes have identical or overlapping recognition sequences, protecting the host DNA from endonuclease cleavage due to the methylation of the recognition site by methyltransferase. In this way, the endonuclease quickly fragments incoming DNA, which does not have the same methylation pattern as the host. Four types of RMS are known, of which type II is the most prevalent in nature. Type II RMS consists of two separate proteins with either an endonuclease or methyltransferase activity and can act independent of each other [13,14].

Until now, only the RMS of *Bbss* and *Borrelia hermsii* have been partially described [11,14]. In the case of *B. afzelii* and *B. garinii,* some studies have been conducted. However, none of these identify and characterize the RMS of these two species [15]. The molecular modification of these two spirochetes remains challenging in the lab. Transformation studies have been attempted, but low transformation efficiencies have repeatedly been observed for low-passage strains [16,17]. We hypothesize that the RMS system of these species confers a barrier to the introduction of DNA and thus prevents genetic modification, as also suggested by the studies of Kawabata et al., 2004 [11] and James et al., 2016 [10]. Therefore, this study is aimed to identify and characterize the RMS in *B. afzelii* and *B. garinii* to circumvent this system and improve transformation efficiency.

## 2. Results

### 2.1. Identification of RMS Genes in Borrelial Patient Isolates

REBASE analysis of Lyme borreliosis species *B. afzelii* and *B. garinii* revealed eight genes that could encode type II RMS proteins. These were *bafPKo_H0010*, *bafPKo_Q0015*, *bafPKo_AA003,* and *bafPKo_I0015* of *B. afzelii* and *bgaPBR_F0006*, *bgaPBR_H0006*, *bgaPBR_K0029,* and *bgaFAR04_F0013* of *B. garinii*.

BLAST analysis identified high similarity (93.6%) of the genes *bafPKo_H0010* and *bgaPBr_K0029* with *bbe02* identified in *Bbss* (Figure S1), indicating that this RMS gene is conserved among Lyme borreliosis spirochetes, while the other genes only share up to 60% similarity.

For *B. afzelii*, we analysed 72 patient isolates by qPCR to assess the prevalence of the identified potential RMS genes. The results are shown in Table S1. For *B. garinii,* all four RMS genes were detected within the four patient isolates that we had for analysis. Two isolates were positive for two genes (*bgaPBR_H0006* and *bgaPBR_K0029*), and the other two isolates were positive for three potential RMS genes (Isolate1: *bgaPBR_H0006*, *bgaFAR04_F0013,* and *gaPBR_K0029*, and Isolate 2: *bgaPBR_F0006*, *bgaPBR_H0006,* and *bgaPBR_K0029*).

### 2.2. Confirmation of the Enzymatic Activity of the RMS Proteins

**Figure 1:**
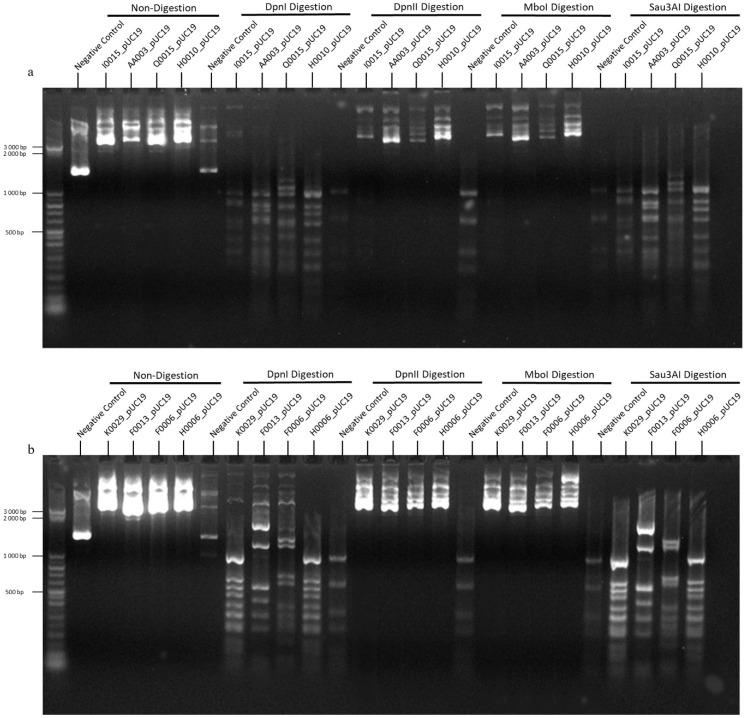
Digestion with methylation-sensitive restriction enzymes to prove methyltransferase activity. (**a**) Digestion of *B. afzelii* genes, I0015, AA003, Q0015 and H0010. (**b**) Digestion of *B. garinii* genes, K0029, F0013, F0006 and H0006. Negative control pUC19 and RMS genes were extracted from a *dam*^−^/*dcm*^−^ *E. coli* strain and subjected to digestion with DpnI, DpnII, MboI and Sau3AI. DpnI cuts GATC sequences that are adenine methylated, DpnI and MboI cut GATC sequences that are adenine nonmethylated, and Sau3AI cuts GATC sequences cytosine nonmethylated.

Plasmids isolated after the expression of RMS genes from *B. afzelii* (Figure 1a) and *B. garinii* (Figure 1b) in the *dam^−^*/*dcm^−^ Escherichia coli* cells produced restriction fragment profiles indicative of adenine methylation at GATC sites. The unmethylated negative control showed an expected opposite restriction fragment profile indicative of the absence of methylation.

**Figure 2:**
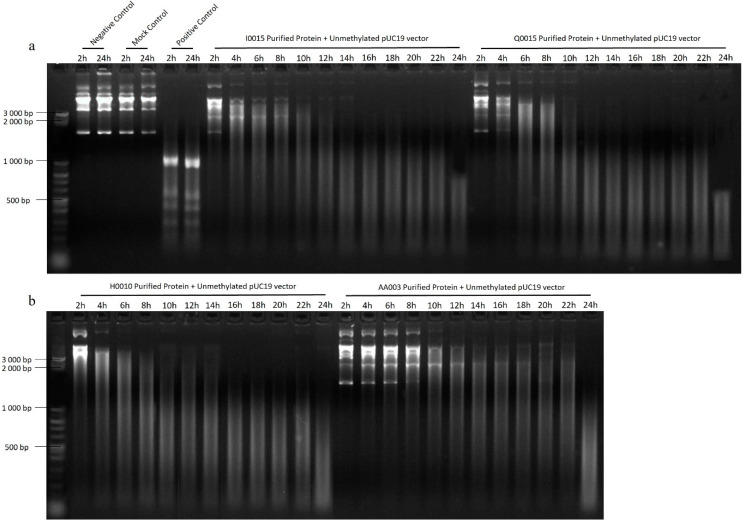
Incubation of *B. afzelii* RMS pure proteins with unmethylated pUC19 vector for 24 h to check endonuclease activity. The negative control and the mock control show no digestion after 24 h, demonstrating that nothing in the incubation mixture without purified RMS proteins can degrade DNA. Positive control, pUC19, was digested with DpnI. Vector pUC19 was incubated with each pure protein of the RMS of *B. afzelii*, I0015, Q0015 (**a**) H0010 and AA003 (**b**) for 24 h. A 50 bp DNA step-ladder from Sigma-Aldrich (Sigma-Aldrich, Vienna, Austria) was used.

**Figure 3:**
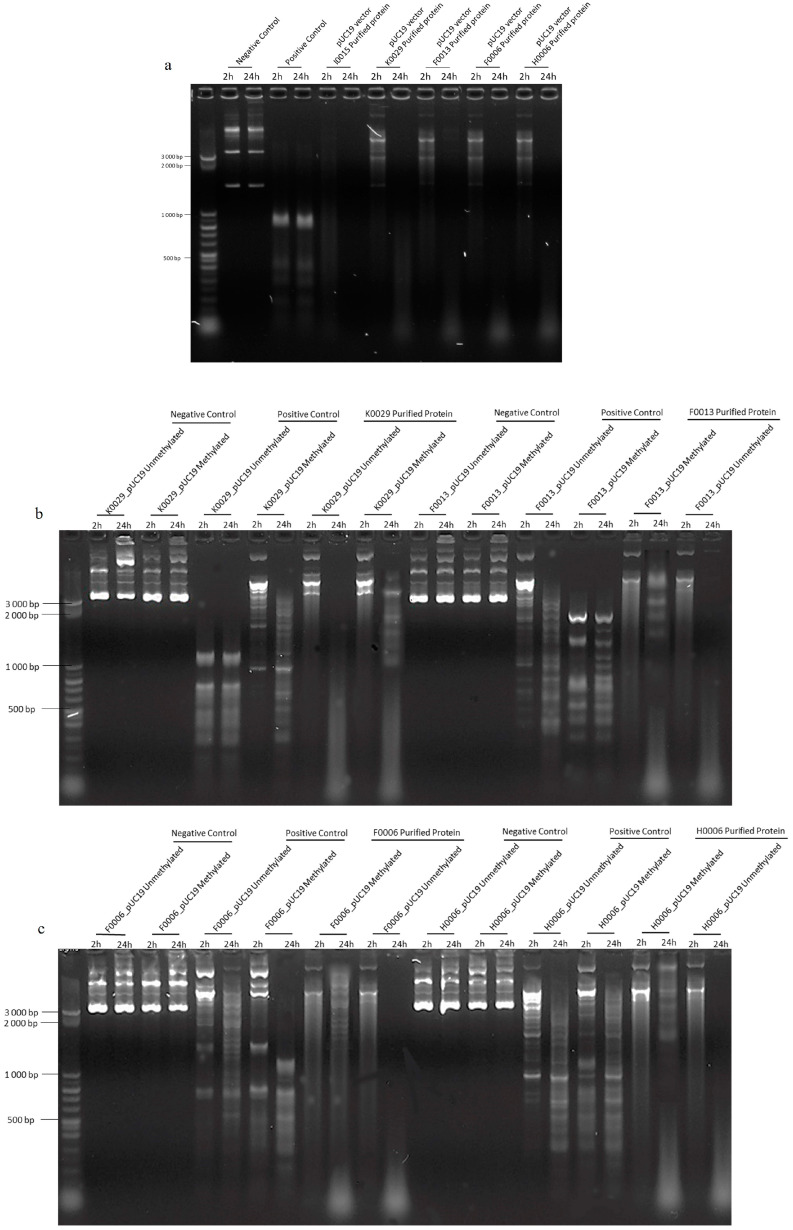
Incubation of purified *B. garinii* RMS proteins with pUC19 vector, respective methylated and unmethylated plasmid to check endonuclease activity and methylation protection against endonuclease activity. (**a**) Negative control, pUC19 was incubated for 24 h in a digestion buffer. Positive control, pUC19 was incubated for 24 h in a digestion buffer with MboI. pUC19 was incubated with the 4 RMS proteins of *B. garinii*, K0029, F0013, F0006 and H0006. (**b**,**c**). Negative control, methylated and unmethylated plasmids were incubated with only digestion buffer for 24 h. Positive controls, methylated and unmethylated plasmids, were incubated with digestion buffer and MboI enzyme for 24 h. Unmethylated and methylated RMS plasmids of *B. garinii*, K0029, F0013, F0006, and H0006 were digested with the respective RMS pure protein for 24 h. A 50 bp DNA step-ladder from Sigma-Aldrich was used.

Endonuclease activity confirmation was observed by incubation of unmethylated pUC19 with 10 mg/mL purified RMS proteins. After 2 h, incomplete digestion was observed. However, after 24 h, pUC19 was completely digested by the *B. afzelii* RMS (Figure 2a,b) and the *B. garinii* RMS (Figure 3a) proteins, indicating specific endonuclease activity.

**Figure 4:**
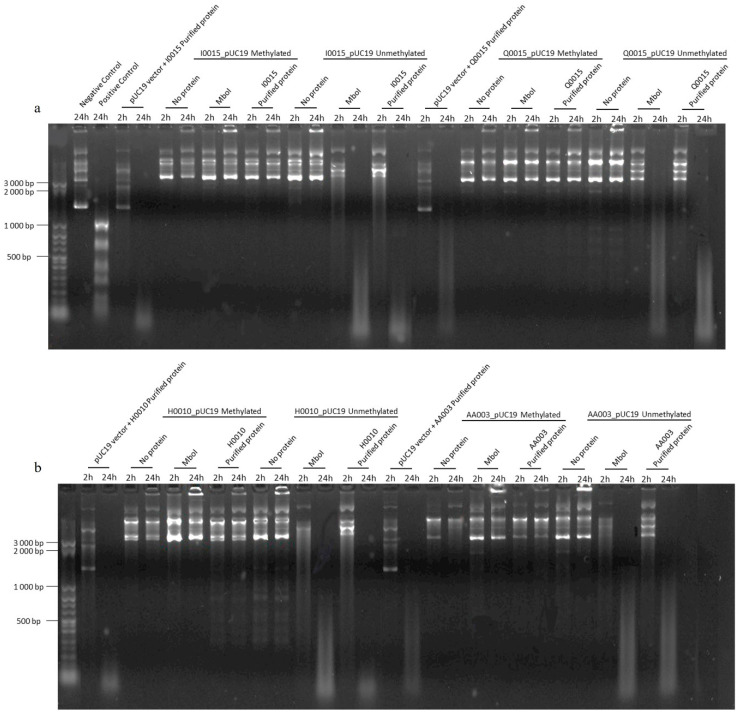
Incubation of purified RMS proteins of *B. afzelii* ((**a**)—I0015 and Q0015; (**b**)—H0010 and AA003) with the respective methylated and unmethylated plasmid to verify methylation protection against endonuclease activity. The negative control, pUC19, was incubated with the protein-free digestion buffer for 24 h. In the positive control, pUC19 was incubated with a digestion buffer and MboI. Vector pUC19 was incubated with all RMS proteins for 24 h as a control of digestion. Methylated and unmethylated RMS plasmids were incubated for 24 h in a digestion buffer without protein as a negative control, incubated for 24 h in a digestion buffer with MboI enzyme as a positive control and incubated for 24 h in a digestion buffer with the respective pure RMS protein. A 50 bp DNA step-ladder from Sigma-Aldrich was used.

After proving the endonuclease activity of the RMS, we assessed if methylation confers protection against this activity. We incubated the purified protein for 24 h with the respective methylated (IPTG-induced) and unmethylated (uninduced) RMS plasmids. After 24 h, all unmethylated plasmids showed DNA digestion using RMS proteins of *B. afzelii* (Figure 4a,b). Meanwhile, adenine methylation protects the methylated plasmid from digestion, as no digestion is observed (Figure 4a,b). However, in the case of *B. garinii*, only a discrete fragmentation of the methylated plasmid was observed (Figure 3b,c), indicative of partial protection. In all cases, the negative controls showed no digestion.

### 2.3. Transformation Efficiency

**Figure 5:**
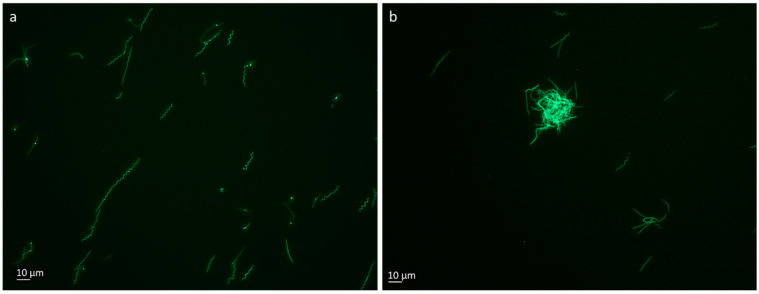
Fluorescence of spirochetes transformed with GFP plasmids. (**a**) *B. burgdorferi* B31 transformed with in vitro methylated pBSV2_OspA_GFP and grown in a modified BSK II medium at 34 °C. (**b**) *B. afzelii* patient isolate was transformed with in vitro methylated pBSV2_OspA_GFP and grown in the same conditions. Both photos were taken using a Nikon Eclipse TE300 Inverted Fluorescence Phase Contrast Microscope (Nikon GmbH, Vienna, Austria) using a 40× objective.

To investigate the influence of the RMS on the transformation efficiency, we methylated the Borrelia transformation vector pBSV2_OspA_GFP. This plasmid expresses a green fluorescence protein regulated by a *Borrelia* promoter [18]. We transformed the methylated and the unmethylated plasmid into *B. burgdorferi* B31 electrocompetent cells in duplicate. A difference in transformation efficiency was observed. When the plasmid was methylated prior, we observed 3.5 ± 1.5 of positive wells out of 192. In comparison, with the unmethylated plasmid, 0.5 ± 0.5 positive wells out of 192 were observed. In the viability control, 25 out of 192 wells showed borrelial growth. Additionally, we successfully transformed a *B. afzelii* (low passage) patient isolate, confirmed by the green fluorescence observed spirochetes (Figure 5).

## 3. Discussion

Previous studies reported RMS genes found in relapsing fever *Borrelia* spp. and Lyme *Borrelia* spp. In a study by Rego et al., 2011 [7], they demonstrated for the first time that the *bbe02* and *bbq67* gene products both modify endogenous DNA and restrict exogenous DNA lacking similar modification in a sequence-specific fashion, as predicted for RMS.

In a study focused on the relapsing fever spirochete *B. hermsii*, a putative methyltransferase gene was observed at the chromosomal locus *bh0463A* [14]. They analyzed the RMS gene similarly to our study and found evidence that it is an adenine-specific methyltransferase.

The RMS of *Bbss* and some relapsing fever borreliae have been previously studied [5,6,7,11,14,15]. However, there is little knowledge about the RMS of *B. afzelii* and *B. garinii*. Therefore, we focused on identifying and characterizing the RMS of these two species.

We computationally predicted four RMS genes in *B. afzelii* and four genes in *B. garinii*, all of which are located on different linear plasmids. The number of RMS genes identified for the borrelial species investigated in our study was higher than those identified in *Bbss* B31 [7] and *B. hermsii* [14]. We hypothesize that habitat, geographical region, host, and tick species are factors that impact the genetic diversity of certain *Borrelia* species. Especially as spirochetes can adapt and survive in different host species due to alterations in gene expression [19]. In a study by Król et al., 2022 [19], they analysed the prevalence of *Bbsl* in ticks and small mammals from central Germany. They observed that *B. afzelii* and *B. garinii* were the most predominant species in both ticks and small mammals, whereas other species were only found in ticks [19]. This led us to the belief that the higher number of RMS genes of both species could be related to their higher prevalence in different tick species and mammals and the necessity to adapt rapidly to these different environments; in contrast to, for example, *B. hermsii*, which has limited tick vectors and hosts [20].

In terms of the location of the genes in the genome, we could see a large difference between Lyme borreliae and relapsing-fever *Borrelia* species. We observed that the RMS genes of some Lyme borreliosis *Borrelia* spp. (i.e., *B. afzelii*, *B. garinii*, *Bbss, Borrelia mayonii,* and *Borrelia bissettii*) were all located on plasmids, and the number of genes varied between three and four per species. On the other hand, relapsing-fever borreliae (i.e., *Borrelia duttonii*, *B. hermsii*, *Borrelia hispanica, Borrelia miyamotoi*, *Borrelia parkeri*, and *Borrelia turicatae*) have a methyltransferase encoding gene, without endonuclease activity, on the chromosome and a type II RMS gene on one of the plasmids that convey both methyltransferase and endonuclease activity.

After analysing the presence of RMS genes in 72 *B. afzelii* and four *B. garinii* patient isolates, we observed that all the isolates studied had at least one of the genes. In our study, we could see a high degree of heterogeneity and plasticity in terms of genetic makeup, as is seen in other studies [21,22]. Since every tested isolate had at least one RMS gene variant present, we believe that the RMS genes are important for the survival of borreliae. This makes it even more noteworthy that all RMS genes discovered in *Bbsl* spirochetes are on plasmids, not the chromosome, as seen in relapsing fever *Borrelia*. On the other hand, plasmids might be more efficiently transferred horizontally between borrelial species or strains. However, the high sequence diversity observed in our study would dispute the horizontal RMS gene transmission between *Bbsl* species.

The resulting restriction fragment profiles of all eight genes were consistent with those reported by James et al., 2016 [14] and provided strong evidence that all genes encode an adenine-specific methyltransferase, confirming the REBASE prediction. DNA methylation is known to play a role in the virulence of other pathogens [23]. The presence of these methyltransferases in *B. afzelii* and *B. garinii* led to the hypothesis that the gene product plays a role in host pathogenicity. In a study by Nye et al., 2019 [24], investigating *Streptococcus pyogenes* RMS, they demonstrated that *S. pyogenes* RMS regulates the expression of gene networks that are important for the virulence of this bacterium.

In a study by Klapatch et al., 1996 [25], they showed that crude cell extracts of *Clostridium thermocellum* exhibit sequence-specific restriction endonuclease activity when incubated with nonmethylated plasmids. However, when incubated with methylated plasmids, the methylation patterns conferred protection against endonuclease digestion. In our study, we observed the total digestion of the plasmid vector when incubated for 24 h with individual, purified proteins from the eight identified RMS genes. This supports our hypothesis that the identified RMS genes have endonuclease activity.

Furthermore, type II RMS use methyltransferases to protect the host genome, while the endonuclease cleaves foreign DNA lacking the specific methylation pattern [13]. We incubated the purified RMS proteins with methylated and unmethylated plasmids to prove this protection of the identified RMS genes in our study. Doing so revealed that the methylated plasmids were unaffected by the respective endonuclease activity, while the unmethylated plasmids were digested entirely within 24 h of incubation. Thereby confirming that the identified RMS genes carry methylation-based protection to endogenous DNA against the endonuclease.

Our research on the transformation of *Borrelia* bacteria has shown a substantial increase in transformation efficiency when the plasmid DNA is methylated prior to transformation. After transformation, we found that 13% (25/192) of the spirochetes were viable in the viability control. Normalising our transformation efficiency results using this general viability of the spirochete strains after electroporation transformation, we extrapolated that 14% (3.5/25) of the viable spirochetes were successfully transformed when a methylated plasmid was used, compared to only 2% (0.5/25) for the unmethylated plasmid. This indicates that methylation greatly improves the transformation efficiency in borrelial strains.

We hypothesize that the increase in transformation efficiency observed by using in vitro methylated plasmids is due to the plasmid being recognized by the RMS of the borrelial strain as host DNA and, thus, is not degraded by the endonuclease. More research will be needed to test the defense efficacy of the identified RMSs against bacteriophages, as well as the role that these RMS genes play in the transformation efficiency of borreliae and the suspected reason for the low transformation efficiency in *Borrelia* species observed [6]. In a study by Kawabata, 2004, they constructed a highly transformable *B. burgdorferi* B31 by inactivating the RMS genes in the linear plasmids, lp25 and lp56. The transformation efficiency with the shuttle vector was increased from < 1 to close to 10 colonies per µg of DNA [11]. This study supports our hypothesis that the RMS plays a crucial role in the transformation efficiency of borreliae.

## 4. Materials and Methods

### 4.1. Borrelial Cultures and DNA Extraction

Brian Crowe and TAKEDA (Vienna, Austria) kindly donated borrelial patient isolates. These isolates were cultured in a modified BSK II medium [26]. After reaching the plateau phase, 1.5 mL of the bacterial suspension was centrifuged at 21,000× *g* for 60 min. DNA was extracted from the resulting pellet using the NucleoSpin Tissue kit (Macherey-Nagel, Düren, Germany). Briefly, the bacterial pellet was re-suspended in 180 μL buffer T1 and 25 μL of proteinase K and incubated under continued shaking (350 rpm) using a thermal shaker, Ditabis MHR 23 (Ditabis, Pforzheim, Germany) at 56 °C for 30 min. The remaining DNA extraction steps were performed as described in the manual provided with the kit.

### 4.2. Identification of Hypothetical Restriction-Modification System Genes

We predicted the RMS genes in *B. afzelii* and *B. garinii* using the restriction enzyme database website (http://rebase.neb.com; accessed on 8 September 2021). For each species, BLAST analysis (accessed on 9 September 2021) of hypothetical RMS genes was performed against all available borrelial genomes.

### 4.3. Detection and Amplification of RMS Genes

We screened 72 of *B. afzelii* and four of *B. garinii* isolates for the presence of the respective RMS genes. To this end, we designed real-time PCR primers using Primer3web (https://primer3.ut.ee/) with the settings published by Thornton et al., 2010 [27] (Table S2) for each gene. The PCR reaction (15 µL total volume) contained 7.5 µL of 2× GoTaq^®^ qPCR Master Mix (Promega, Vienna, Austria) containing BRYT green, 0.4 µM of each primer, 0.2 µL CXR reference dye (Promega), 1 µL of template DNA, and PCR-grade water (Sigma-Aldrich, Vienna, Austria). The qPCRs were performed using the following protocol: 95 °C for 2 min, followed by 45 cycles of 95 °C for 15 s, and 60 °C for 1 min.

All putative RMS genes were detected in one or more isolates using the qPCRs described above. Additional primers were designed to amplify the whole gene with added BamHI and KpnI recognition sites for *B. afzelii* primers, KpnI, and SalI restriction sites for *B. garinii* (Table S2). The PCRs were performed with Phire Hot Start II polymerase (Thermo Fisher Scientific, Vienna, Austria) using 5 µL Phire reaction buffer, 200 nM of each dNTP (Solis Biodyne, Tartu, Estonia), 400 nM of each primer, 0.5 µL Phire II polymerase, 2.5 µL template DNA, PCR-grade water (Sigma-Aldrich) in a total volume of 25 µL. The PCRs were performed using the following protocol: initial 98 °C for 30 s, followed by 45 cycles of 98 °C for 5 s, a gradient annealing temperature between 50 °C and 60 °C for 5 s and 72 °C for 1 min, and a final elongation step at 72 °C for 1 min.

PCR products formed with the highest annealing temperatures were cloned into a pJET1.2 cloning vector using the CloneJET PCR cloning kit (Thermo Fisher Scientific) and DH5α *E. coli* cells according to the manufacturer’s instructions. The plasmids were isolated using the GeneJet Plasmid Miniprep kit (Thermo Fisher Scientific). The insert DNA sequence was confirmed by bidirectional sequencing (Microsynth Austria GmbH, Vienna, Austria).

### 4.4. Cloning and Transformation

The constructed plasmids were digested consecutively with the respective restriction enzymes (New England Biolabs, Frankfurt, Germany), as described by the manufacturer. The digested RMS genes were cloned into the pUC19 vectors, an IPTG inducible expression vector (Thermo Fisher Scientific) and used to transform DH5α *E. coli* cells. Blue and white screening was used to identify positive clones after overnight incubation at 37 °C on LB agar plates containing ampicillin (50 µg/mL), IPTG (1 mM), and X-gal (20%).

From the overnight culture plates, three white colonies were selected and grown overnight in an LB medium containing ampicillin (50 µg/mL). Subsequently, the plasmids of the cultures were extracted using the GeneJet Plasmid Miniprep kit (Thermo Fisher Scientific) as described in the manual. The insert DNA sequence was verified by sequencing (Microsynth Austria GmbH). Finally, all obtained plasmids were used to transform a *dam^-^/dcm^-^ E. coli* strain (C2925I, New England Biolabs) and plated out on ampicillin-containing LB agar plates (50 µg/mL).

### 4.5. Gene Expression

From *dam^−^/dcm^−^ E. coli* plates grown overnight, four colonies were picked and subcultured overnight in LB medium containing ampicillin (50 µg/mL). The next day, the liquid culture was diluted 1:50 in 5 mL LB medium with ampicillin (50 µg/mL) and incubated at 37 °C in a shaking incubator (180 rpm) until the optical density of 600 nm (OD_600_) reached 0.6. Subsequently, the genes of interest were induced using IPTG (1 mM) for 4 h at 37 °C with agitation (180 rpm). Finally, the potentially methylated plasmids were extracted using the GeneJet Plasmid Miniprep kit (Thermo Fisher Scientific). A mock control consisted of the *E. coli* strain transformed with a pUC19 plasmid without an insert.

### 4.6. In Vivo Confirmation of Methylase Activity

Following plasmid extraction, 500 ng of the constructed plasmids were digested with DpnI, DpnII, MboI, or Sau3AI (New England Biolabs) following the manufacturer’s protocol. DpnI cleaves adenine-methylated GATC sites, and DpnII and MboI cleave only unmethylated GATC sites. Sau3AI cleaves all GATC sites regardless of adenine methylation but is impeded by methylated cytosine. The resulting DNA fragments were resolved by agarose gel electrophoresis.

### 4.7. In Vivo Confirmation of Endonuclease Activity

The RMS genes were cloned into pRSETC vectors (Thermo Fisher Scientific) using the same restriction enzymes used before and transformed into DH5α *E. coli* cells. The plasmids were extracted with a GeneJet Plasmid Miniprep kit (Thermo Fisher Scientific) and sent for sequencing (Microsynth Austria GmbH).

Verified plasmids were used to transform *E. coli* BL21 (DE3) pLys cells, which were plated on LB agar plates containing ampicillin (50 µg/mL) and chloramphenicol (50 µg/mL).

The colonies resulting were harvested and inoculated in LB medium with ampicillin (50 µg/mL) and chloramphenicol (50 µg/mL) at 37 °C with agitation (180 rpm) and incubated overnight at 37 °C. Subsequently, the culture was diluted 1:50 in 250 mL of pre-warmed LB medium with ampicillin (50 µg/mL) and chloramphenicol (50 µg/mL) and incubated at 37 °C with agitation (180 rpm) until the OD_600_ was between 0.5 and 0.7. Protein expression was induced using IPTG (1 mM) for 4 h. The cells were harvested and frozen at −20 °C overnight.

The cell pellet was re-suspended in 5 mL lysis buffer (50 mM NaH_2_PO_4_, 300 mM NaCl and 10 mM Imidazole) and lysed by three freezing and thawing cycles. Subsequently, the cells were sonicated on ice (6 × 10 s with 10 s pauses at 70% strength) using a model 120 Sonic Dismembrator (Fisher Scientific, Vienna, Austria). The suspension was centrifuged at 10,000× *g* at 4 °C for 30 min, and from the supernatant, the 6×His-tagged proteins were purified using Ni-NTA Agarose (Qiagen, Hilden, Germany) following the protocol provided.

To confirm the potential endonuclease activity and the methylation-depended protection thereof, 500 ng of methylated or unmethylated pUC19, respectively, was combined with 10 mg/mL purified RMS proteins in a digestion buffer (10 mM Tris-HCl pH 8.0; 1 mM EDTA pH 8.0; 50 mM NaCl, 10 mM MgCl_2_, 1% Triton X-100 and nuclease-free water) in a total volume of 260 µL. This mixture was divided into twelve tubes, each containing 20 µL, and then incubated for 24 h at 37 °C. Every 2 h, a tube was taken and resolved using agarose gel electrophoresis. The mock control consisted of purified protein extracts from BL21 (DE3) pLys transformed with an empty pRSETC vector. We used methylated and unmethylated pUC19 as controls. For negative control, we incubated pUC19 with 5 µL of digestion buffer. For positive control, we incubated the samples with 5 µL of digestion buffer and 1 µL of DpnI (20,000 units/mL). To check for methylation-based protection, we used 1 µL of MboI (5000 units/mL) instead.

### 4.8. Transformation Efficiency Analysis

To investigate the transformation efficiency, a methylated pBSV2_OspA_GFP [18] plasmid was obtained by co-transformation into a *dam^−^/dcm^−^ E. coli* strain containing RMS for *Borrelia*. The methylated pBSV2_OspA_GFP was used to transform *B. burgdorferi* B31 and a *B. afzelii* patient isolate. Unmethylated pBSV2_OspA_GFP was used as a control. Preparation of electrocompetent *B. burgdorferi* B31 and *B. afzelii* patient isolate cells and transformation were carried out as described by Samuels 1995 [28]. After electroporation, cells were resuspended in 1 mL of prewarmed modified BSK II medium and incubated for 20 h at 34 °C. A dilution of 1 spirochete per well was plated in each well of a 96-well plate with kanamycin (50 µg/mL), and in the viability control, no antibiotics were added to the wells. Plates were incubated at 34 °C for 14 days. Colony counts (i.e., wells with spirochete growth) were used to determine the transformation efficiency for each isolate. Transformation efficiency is expressed as the ratio between the number of colonies grown in antibiotic-containing wells originating from methylated and unmethylated plasmids, normalised by the viability controls.

## 5. Conclusions

In conclusion, our study demonstrates that bypassing the RMS defenses is necessary to increase transformation efficiency. The knowledge gained about the RMS of these *Borrelia* species will allow us to increase the transformation success rate of low passage infectious borrelial (patient) isolates and enable further host-pathogen and tick interaction studies. So far, we have been one of the few laboratories able to modify patient isolates successfully. Additionally, our results could potentially contribute to the development of targeted treatment for borreliosis through phage therapy. Phage modification to convey host methylation patterns can be used to develop and improve phage therapy, which has been investigated in previous years [29,30]. This could reduce the reliance on antibiotics in the future.

## Data Availability

Data is contained within the article or Supplementary Materials.

## Supplementary Materials

The following supporting information can be downloaded at: https://www.mdpi.com/article/10.3390/ijms252111343/s1.

## References

[1] Marques A.R., Strle F., Wormser G.P. (2021). Comparison of Lyme disease in the United States and Europe. Emerg. Infect. Dis..

[2] Stanek G., Wormser G.P., Gray J., Strle F. (2012). Lyme borreliosis. Lancet.

[3] Kurokawa C., Lynn G.E., Pedra J.H.F., Pal U., Narasimhan S., Fikrig E. (2020). Interactions between *Borrelia burgdorferi* and ticks. Nat. Rev. Microbiol..

[4] Schwartz I., Margos G., Casjens S.R., Qiu W.G., Eggers C.H. (2021). Multipartite genome of Lyme disease *Borrelia*: Structure, variation and prophages. Curr. Issues Mol. Biol..

[5] Chan K., Alter L., Barthold S.W., Parveen N. (2015). Disruption of *bbe02* by insertion of a luciferase gene increases transformation efficiency of *Borrelia burgdorferi* and allows live imaging in Lyme disease susceptible C3H mice. PLoS ONE.

[6] Lawrenz M.B., Kawabata H., Purser J.E., Norris S.J. (2002). Decreased electroporation efficiency in *Borrelia burgdorferi* containing linear plasmids lp25 and lp56: Impact on transformation of infectious *B. burgdorferi*. Infect. Immun..

[7] Rego R.O., Bestor A., Rosa P.A. (2011). Defining the plasmid-borne restriction-modification systems of the Lyme disease spirochete *Borrelia burgdorferi*. J. Bacteriol..

[8] Margos G., Hepner S., Mang C., Marosevic D., Reynolds S.E., Krebs S., Sing A., Derdakova M., Reiter M.A., Fingerle V. (2017). Lost in plasmids: Next generation sequencing and the complex genome of the tick-borne pathogen *Borrelia burgdorferi*. BMC Genomics.

[9] Grimm D., Eggers C.H., Caimano M.J., Tilly K., Stewart P.E., Elias A.F., Radolf J.D., Rosa P.A. (2004). Experimental assessment of the roles of linear plasmids lp25 and lp28-1 of *Borrelia burgdorferi* throughout the infectious cycle. Infect. Immun..

[10] Norris S.J., Howell J.K., Odeh E.A., Lin T., Gao L., Edmondson D.G. (2011). High-throughput plasmid content analysis of *Borrelia burgdorferi* B31 by using luminex multiplex technology. Appl. Environ. Microbiol..

[11] Kawabata H., Norris S.J., Watanabe H. (2004). BBE02 disruption mutants of *Borrelia burgdorferi* B31 have a highly transformable, infectious phenotype. Infect. Immun..

[12] Norris S.J., Carter C.J., Howell J.K., Barbour A.G. (1992). Low-passage-associated proteins of *Borrelia burgdorferi* B31: Characterization and molecular cloning of *OspD*, a surface-exposed, plasmid-encoded lipoprotein. Infect. Immun..

[13] Vasu K., Rao D.N., Nagaraja V. (2019). Restriction-Modification Systems.

[14] James A.E., Rogovskyy A.S., Crowley M.A., Bankhead T. (2016). Characterization of a DNA adenine methyltransferase gene of *Borrelia hermsii* and its dispensability for murine infection and persistence. PLoS ONE.

[15] Wachter J., Martens C., Barbian K., Rego R.O.M., Rosa P. (2021). Epigenomic landscape of Lyme disease spirochetes reveals novel motifs. mBio.

[16] Bontemps-Gallo S., Lawrence K.A., Richards C.L., Gherardini F.C. (2018). Genomic and phenotypic characterization of *Borrelia afzelii* BO23 and *Borrelia garinii* CIP 103362. PLoS ONE.

[17] Fingerle V., Goettner G., Gern L., Wilske B., Schulte-Spechtel U. (2007). Complementation of a *Borrelia afzelii* OspC mutant highlights the crucial role of OspC for dissemination of *Borrelia afzelii* in *Ixodes ricinus*. Int. J. Med. Microbiol..

[18] Stewart P.E., Thalken R., Bono J.L., Rosa P. (2001). Isolation of a circular plasmid region sufficient for autonomous replication and transformation of infectious *Borrelia burgdorferi*. Mol. Microbiol..

[19] Król N., Obiegala A., Imholt C., Arz C., Schmidt E., Jeske K., Ulrich R.G., Rentería-Solís Z., Jacob J., Pfeffer M. (2022). Diversity of *Borrelia burgdorferi* sensu lato in ticks and small mammals from different habitats. Parasit. Vectors.

[20] Stewart P.E., Raffel S.J., Gherardini F.C., Bloom M.E. (2022). Kinetics of tick infection by the relapsing fever spirochete *Borrelia hermsii* acquired through artificial membrane feeding chambers. Sci. Rep..

[21] Becker N.S., Rollins R.E., Nosenko K., Paulus A., Martin S., Krebs S., Takano A., Sato K., Kovalev S.Y., Kawabata H. (2020). High conservation combined with high plasticity: Genomics and evolution of *Borrelia bavariensis*. BMC Genom..

[22] Takacs C.N., Wachter J., Xiang Y., Karaboja X., Ren Z., Scott M., Stoner M.R., Irnov I., Jannetty N., Rosa P.A. (2022). Polyploidy, regular patterning of genome copies, and unusual control of DNA partitioning in the Lyme disease spirochete. bioRxiv.

[23] Seib K.L., Srikhanta Y.N., Atack J.M., Jennings M.P. (2020). Epigenetic regulation of virulence and immunoevasion by phase-variable restriction-modification systems in bacterial pathogens. Annu. Rev. Microbiol..

[24] Nye T.M., Jacob K.M., Holleyid E.K., Nevarez J.M., Dawidid S., Simmons L.A., Watson M.E. (2019). DNA methylation from a type I restriction modification system influences gene expression and virulence in *Streptococcus pyogenes*. PLoS Pathog..

[25] Klapatch T.R., Demain A.L., Lynd L.R. (1996). Restriction endonuclease activity in *Clostridium thermocellum* and *Clostridium thermosaccharolyticum*. Appl. Microbiol. Biotechnol..

[26] Reiter M., Schötta A.-M.M., Müller A., Stockinger H., Stanek G. (2015). A newly established real-time PCR for detection of *Borrelia miyamotoi* in *Ixodes ricinus* ticks. Ticks Tick. Borne Dis..

[27] Thornton B., Basu C. (2011). Real-time PCR (qPCR) primer design using free online software. Biochem. Mol. Biol. Educ..

[28] Samuels D.S. (1995). Electrotransformation of the spirochete *Borrelia burgdorferi*. Methods Mol. Biol..

[29] Eggers C.H., Gray C.M., Preisig A.M., Glenn D.M., Pereira J., Ayers R.W., Alshahrani M., Acabbo C., Becker M.R., Bruenn K.N. (2016). Phage-mediated horizontal gene transfer of both prophage and heterologous DNA by φBB-1, a bacteriophage of *Borrelia burgdorferi*. Pathog. Dis..

[30] Jernigan D.A., Hart M.C., Dodd K.K., Jameson S., Farney T. (2021). Induced native phage therapy for the treatment of Lyme disease and relapsing fever: A retrospective review of first 14 months in one clinic. Cureus.

