# Supplementary material for: Optimising Transformation Efficiency in Borrelia: Unravelling the Role of the Restriction-Modification System of Borrelia afzelii and Borrelia garinii
Source: Int J Mol Sci. 2024 Oct 22;25(21):11343. doi: 10.3390/ijms252111343 (PMC11546952; doi:10.3390/ijms252111343)
Supplement: Supplementary file 1 [file ijms-25-11343-s001.zip › ijms-3254995-supplementary.pdf]

## Supplement

### Supplemental Table S1. Prevalence of *B. afzelii* and *B. garinii* RMS genes in patient isolates.

| RMS genes | #1023 | #1061 | #1176 | #1192 | #1193 | #1205 | #1207 | #1217 | #1225 | #1230 | #1231 | #1232 | #1235 | #1237 | #1035 | #1039 | #1007 | #1033 | #1053 | #1179 | #1224 | #1229 | #1234 | #1236 |
|---|---|---|---|---|---|---|---|---|---|---|---|---|---|---|---|---|---|---|---|---|---|---|---|---|
| *bafPKo_I0015* | ■ | ■ | ■ | ■ | ■ | ■ | ■ | ■ | ■ | ■ | ■ | ■ | ■ | ■ | | | ■ | ■ | ■ | ■ | ■ | ■ | ■ | ■ |
| *bafPKo_Q0015* | | | | | | | | | | | | | | | | | | | | | | | | |
| *bafPKo_AA003* | | | | | | | | | | | | | | | | | ■ | ■ | ■ | ■ | ■ | ■ | ■ | ■ |
| *bafPKo_H0010* | | | | | | | | | | | | | | | ■ | ■ | | | | | | | | |
| | #1022 | #1038 | #1052 | #1180 | #1214 | #1187 | #1233 | #1004 | #1005 | #1006 | #1034 | #1054 | #1056 | #1059 | #1177 | #1178 | #1181 | #1183 | #1185 | #1186 | #1188 | #1190 | #1191 | #1195 |
| *bafPKo_I0015* | ■ | ■ | ■ | ■ | ■ | ■ | ■ | ■ | ■ | ■ | ■ | ■ | ■ | ■ | ■ | ■ | ■ | ■ | ■ | ■ | ■ | ■ | ■ | ■ |
| *bafPKo_Q0015* | | | | | | ■ | ■ | | | | | | | | | | | | | | | | | |
| *bafPKo_AA003* | | | | | | | | ■ | ■ | ■ | ■ | ■ | ■ | ■ | ■ | ■ | ■ | ■ | ■ | ■ | ■ | ■ | ■ | ■ |
| *bafPKo_H0010* | ■ | ■ | ■ | ■ | ■ | | | ■ | ■ | ■ | ■ | ■ | ■ | ■ | ■ | ■ | ■ | ■ | ■ | ■ | ■ | ■ | ■ | ■ |
| | #1199 | #1200 | #1202 | #1215 | #1218 | #1226 | #1240 | #1241 | #1057 | #1203 | #1216 | #1219 | #1238 | #1204 | #1055 | #1058 | #1175 | #1182 | #1189 | #1201 | #1206 | #1210 | #1220 | #1239 |
| *bafPKo_I0015* | ■ | ■ | ■ | ■ | ■ | ■ | ■ | ■ | ■ | ■ | ■ | ■ | ■ | ■ | ■ | ■ | ■ | ■ | ■ | ■ | ■ | ■ | ■ | ■ |
| *bafPKo_Q0015* | | | | | | | | | ■ | ■ | ■ | ■ | ■ | ■ | ■ | ■ | ■ | ■ | ■ | ■ | ■ | ■ | ■ | ■ |
| *bafPKo_AA003* | ■ | ■ | ■ | ■ | ■ | ■ | ■ | ■ | | | | | | ■ | ■ | ■ | ■ | ■ | ■ | ■ | ■ | ■ | ■ | ■ |
| *bafPKo_H0010* | ■ | ■ | ■ | ■ | ■ | ■ | ■ | ■ | ■ | ■ | ■ | ■ | ■ | | ■ | ■ | ■ | ■ | ■ | ■ | ■ | ■ | ■ | ■ |
| | #1035 | #1039 | #1195 | #1226 | | | | | | | | | | | | | | | | | | | | |
| *bgaPBr_F0006* | | | | ■ | | | | | | | | | | | | | | | | | | | | |
| *bgaPBr_H0006* | ■ | ■ | ■ | ■ | | | | | | | | | | | | | | | | | | | | |
| *bgaPBr_K0029* | ■ | ■ | ■ | ■ | | | | | | | | | | | | | | | | | | | | |
| *bgaFar04_F0013* | ■ | | | | | | | | | | | | | | | | | | | | | | | |

#isolate ID number. In dark grey are marked the positive RMS genes for each patient isolate. We observed for *B. afzelii* 16 isolates positive for one RMS gene, 15 for two RMS genes, 31 for three RMS genes, and 10 for four RMS genes. For *B. garinii,* we observed 2 isolates positive for two RMS genes and 3 isolates positive for three RMS genes.

### Supplemental Table S2. Primers designed and used in this study

| Primer | Sequence (5' to 3') | Genome location | Amplicon size |
|---|---|---|---|
| **Real-time PCR primers for the detection of the RMS genes** | | | |
| BafAA003_fwd_qPCR | AACTTAGCATTGCGATTTGTACAGA | lp28-7 | 81 bp |
| BafAA003_rev_qPCR | CCCTTAGAAGAATGTTGCTTGGAAA | | |
| BafI0015_fwd_qPCR | AACTGACTTTATGGTTGTGTCCTCA | lp28-4 | 97 bp |
| BafI0015_rev_qPCR | ATGACCCGAACCACAAGAATTATCA | | |
| BafH0010_fwd_qPCR | ATGCTGAGTAATTTCTTTGCCTGAT | lp28-3 | 115 bp |
| BafH0010_rev_qPCR | AGAGATACTTTATGAGAGACCCGAC | | |
| BafQ0015_fwd_qPCR | AGAATGCAGTCCACCTAATTACTTT | lp32-10 | 120 bp |

| | | | |
|---|---|---|---|
| BafQ0015_rev_qPCR | TTTACGCCGACACTATATTCATTGA | | |
| Bg_F0006_qPCR_fwd<br>Bg_F0006_qPCR_rev | GAGATGCGACACTACATATGGAGCT<br>TTCTTGACTTTGCAACTGGAACTGG | lp28-9 | 128 bp |
| Bg_F0013_qPCR_fwd<br>Bg_F0013_qPCR_rev | AACTATTAAAGGCGGTGAAGAAGGA<br>TACTCATGCCTCTGAATGTGATGTT | lp28-4 | 87 bp |
| Bg_H0006_qPCR_fwd<br>Bg_H0006_qPCR_rev | TCTAGCTCGTACCATACTTTCTCTGTT<br>ATTGATAATTCTTGTGGGTCAGGGC | lp28-3 | 80 bp |
| Bg_K0029_qPCR_fwd<br>Bg_K0029_qPCR_rev | TCACCAGTATTTCTTCAAGTTCACT<br>AGGATTATTTGCAGAAGACAAGGTT | lp36 | 83 bp |
| PCR primers for the amplification of complete RMS genes* | | | |
| BafAA003_fwd_KpnI<br>BafAA003_rev_BamHI | TTCAGGTACCATCTATTATGCAATCCAA<br>TTCAGGATCCATGCTATGATGAAAACTAA | lp28-7 | 3269 bp |
| BafI0015_fwd_BamHI<br>BafI0015_rev_KpnI | TTCAGGATCCTAATGAAAACTAATGATGTTG<br>TTCAGGTACCTTCCACCCTATTATACTAA | lp28-4 | 3861 bp |
| BafH0010_Fwd_ KpnI<br>BafH0010_Rev_ BamHI | GGGCAGGTACCTTATTTATGATAAAAAATT<br>GCATGGATCCTAATGAAAACTAATGATATCG | lp28-3 | 3868 bp |
| BafQ0015_fwd_BamHI<br>BafQ0015_rev_KpnI | GGCAGGATCCTTATAAAGATGAACAGTAATG<br>GGCTGGTACCTTATTCATTGAATTCTTTAAG | lp32-10 | 3205 bp |
| Bg_F0006_ SalI_fwd<br>Bg_F0006_KpnI_ rev | GCTAGTCGACGGGAAAATATGATGTTATTA<br>GCTAGGTACCTGTGCTAAATACCAAATTC | lp28-9 | 3177 bp |
| Bg_F0013_SalI_fwd<br>Bg_F0013_KpnI_rev | GGCTGGTCGACTAGTTTAGTTGATATTAA<br>GCTAGGTACCTAATTGTATAGCTAAGCTAC | lp28-4 | 3157 bp |
| Bg_H0006_SalI_fwd<br>Bg_H0006_KpnI_ rev | GGCTAGTCGACTGTAAGCATAAATAATGAA<br>GCTAGGTACCGTTAAGCTTAATTAAGCT | lp28-3 | 3861 bp |
| Bg_K0029_SalI_fwd<br>Bg_K0029_KpnI_rev | GGCTAGTCGACTGTAAGCATAAATAATGAA<br>GCTAGGTACCAAGCTTAGTTAAGTTTGTTT | lp36 | 3855 bp |

***Restriction enzyme recognition site is in bold and underlined.**

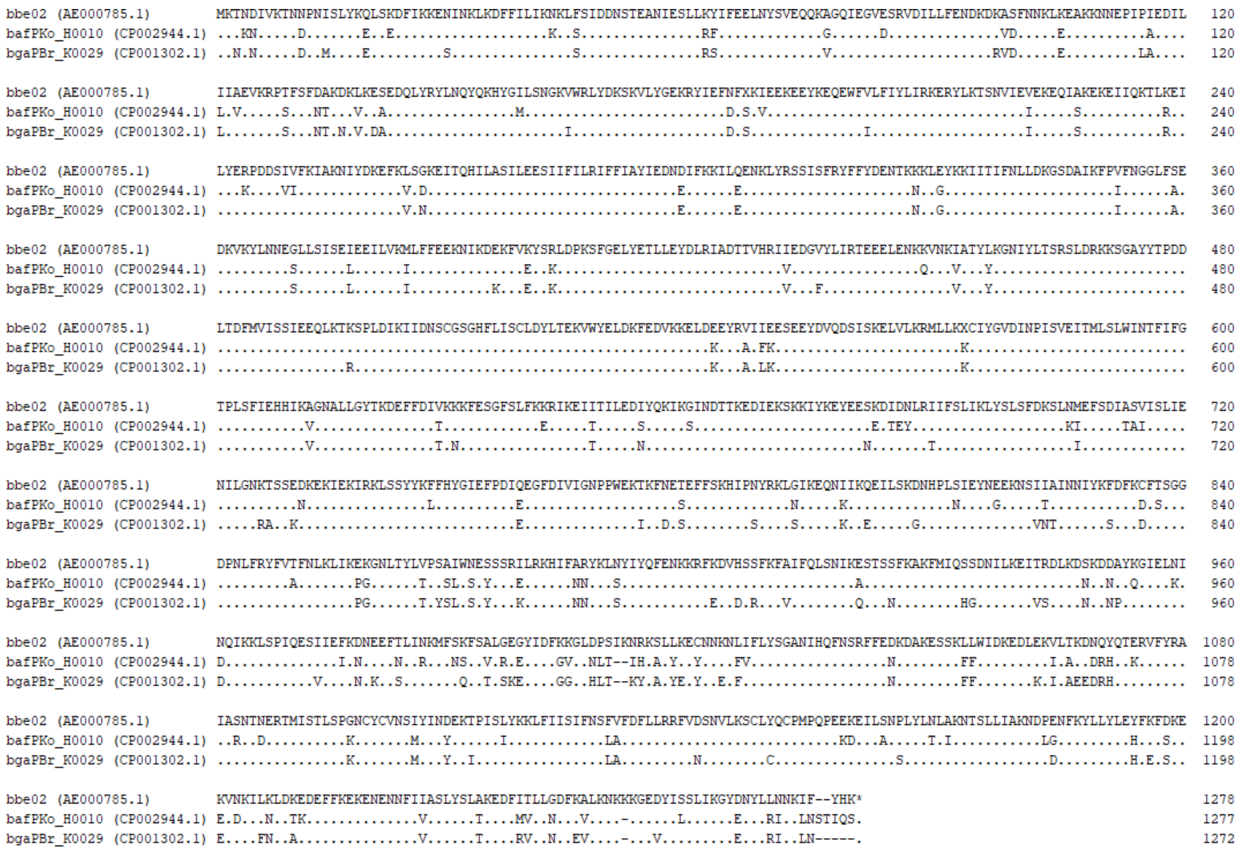


**Figure S1. Alignment, obtained with Geneious Prime® 2023.1.1, of the *bbe02* gene of *Bbss* in. comparison with *bafPKo_H0010* of *B. afzelii* and *bgaPBr_K0029* of *B. garinii*.** Blast analysis identified a 93.6 % similarity between these three genes. Showing that this gene is conserved among Lyme borreliosis spirochetes.
